# Untargeted metabolomics and proteomics reveals cocoa-mediated mitigation of valproic acid-induced dysregulation in a zebrafish model of autism: pilot study

**DOI:** 10.1007/s11306-026-02482-w

**Published:** 2026-06-18

**Authors:** Jeffrey Li, Isra’a Haj-Husein, Nathan Ghafari, Leila Khorraminezhad, Michèle Iskandar, Charlotte Zaouter, Matthias Klein, Lekha Sleno, Shunmoogum A. Patten, Stan Kubow

**Affiliations:** 1 School of Human Nutrition, McGill University, Montreal, Canada; 2 Département de chimie, Université du Québec à Montréal, Montréal, Canada; 3 Institut national de la recherche scientifique, Québec City, Canada; 4 Animal Science, McGill University, Montreal, Canada

**Keywords:** Autism spectrum disorder, Valproic acid, Zebrafish, Cacao, Polyphenols, Metabolomics, Proteomics

## Abstract

**Introduction:**

Autism spectrum disorder (ASD) is a neurodevelopmental condition characterized by behavioral impairments and limited therapeutic options. Emerging evidence suggests that plant-derived polyphenols may offer neuroprotective benefits.

**Objectives:**

This pilot study aimed to investigate the therapeutic potential of polyphenol-rich cocoa extract in a valproic acid (VPA)-induced zebrafish model of ASD.

**Methods:**

Zebrafish were exposed to 3 μM VPA, cocoa powder providing 2.5 μM (–)-epicatechin, a combination of both, or left untreated. Behavioral phenotyping was conducted using DanioVision and gut morphology was assessed. Untargeted metabolomic and proteomic profiling was performed followed by univariate and multivariate analyses.

**Results:**

VPA exposure induced ASD-like behavioral hyperactivity, and severe gastrointestinal abnormalities. Cocoa co-treatment ameliorated both behavioral performance and gut architecture. Metabolomic profiling revealed VPA-associated disruptions in neurotransmission, methylation, mitochondrial function and redox homeostasis. Proteomic profiling showed elevated levels of trafficking protein particle complex subunit 11, proteasomal ubiquitin receptor, betaine-homocysteine S-methyltransferase 1 (BHMT-1), and desmoplakin-A, consistent with genotoxic stress and impaired protein trafficking. Cocoa co-treatment normalized BHMT-1 and desmoplakin-A expression and mitigated broader metabolic dysregulation.

**Conclusion:**

Collectively, these results suggest that polyphenol-rich cocoa may represent a promising multi-targeted nutraceutical approach for mitigating ASD-related neurodevelopmental and metabolic disturbances.

**Supplementary Information:**

The online version contains supplementary material available at https://doi.org/10.1007/s11306-026-02482-w.

## Introduction

Autism spectrum disorder (ASD) is a complex neurodevelopmental condition characterized by social communication deficits, restricted interests and repetitive behaviors (Lord et al., 2018). While genetic factors contribute to ASD etiology, expression of susceptibility is highly variable and influenced by environmental factors (Veenstra-VanderWeele et al., 2004; Wang et al., 2017). Prenatal exposure to agents such as anti-epileptic valproic acid (VPA) and thalidomide drugs increases ASD risk (Wang et al., 2017; Rasalam et al., 2005). Early screening and diagnosis are critical for timely intervention and improved developmental outcomes.

Despite extensive research, ASD mechanisms remain unclear. Recent studies implicate mitochondrial dysfunction and gut microbiota dysbiosis, both affecting neurodevelopmental pathways in ASD (Frye, 2020; Chen et al., 2022). Perturbations in synaptic activity, neurotransmitter signaling, neuroplasticity, and neuroinflammation, linked to imbalances in nitric oxide, serotonin, and glutamate are associated with ASD behavioral phenotypes (Tripathi et al., 2020; Montanari et al., 2022; Rodnyy et al., 2024). Previous proteomic studies also revealed alterations in neuronal connectivity proteins, such as neurexin and neuroligin (Trobiani et al., 2020), mitochondrial enzymes (Siddiqui et al., 2016), and immune response pathways, such as complement C4 and major histocompatibility complex DR beta 1 (Ohja et al., 2018).

Current therapies for ASD are primarily behavioral, imposing financial and time burdens (Politte et al., 2015). Pharmacological options target comorbid symptoms without addressing core deficits in social communication and often cause adverse effects. Consequently, these limitations underscore the need for novel well-tolerated therapeutic strategies (Fung et al., 2016). Among animal models investigating the neurobiological and genetic mechanisms underlying ASD, embryonic exposure of zebrafish to valproic acid (VPA) has emerged as a widely used model (Flores-Prieto et al., 2024). This model reproducibly induces behavioral and developmental alterations characteristic of ASD due to its rapid embryonic development, enabling early assessment of neurodevelopmental perturbation and suitability for multi‑level mechanistic studies. Few studies, however, have integrated metabolomics and proteomics with behavioral outcomes in ASD models.

Flavonoids, a major subclass of polyphenols abundant in cocoa beans, have been increasingly investigated as nutraceuticals with neuroprotective properties. Cocoa is particularly rich in flavan-3-ols, including catechin, epicatechin, and their oligomeric procyanidin derivatives, which represent key bioactive constituents. These compounds have been associated with enhanced neuroplasticity, neurogenesis, and synaptogenesis, and as such carry significant cognitive benefits (Carrera et al., 2025). Evidence suggests that these benefits are mediated, at least in part, through antioxidant and immunomodulatory activities, endothelial vascular responses, and anti-inflammatory mechanisms, all of which are critical for maintaining cognitive function and cerebrovascular health (Nehlig, 2013; Sorond et al., 2013; Gildawie et al., 2018; Potì et al., 2019; Carrera et al., 2025).

In addition, cocoa polyphenols exert prebiotic effects that promote beneficial gut microbiota, thereby modulating the gut–brain axis (Martinez et al., 2017; Plamada & Vodnar, 2021). This bidirectional communication network contributes to cognitive and emotional regulation through complex interactions among the gastrointestinal, neuroendocrine, neuroimmune, and autonomic nervous systems. Disruptions in gut microbiota composition and the resulting dysregulation of the gut–brain axis have been implicated in the pathogenesis of ASD (Morton et al., 2023).

Although VPA-treated zebrafish models and dietary polyphenol interventions are valuable tools for dissecting mechanistic pathways underlying ASD-like phenotypes, integrative multi-omic analyses combining metabolomics, proteomics, behavior, and gut morphology remain limited. This pilot study aimed to evaluate the effects of a polyphenol-rich cocoa extract in a VPA-induced zebrafish model of ASD via multi-omics profiling as well as behavioral and gut morphology assessment. We hypothesized that cocoa polyphenols mitigate the neurodisruptive effects of VPA exposure and attenuate ASD-like phenotypes by restoring redox balance, improving mitochondrial function, modulating neurotransmitter-related metabolic pathways and preserving gut integrity.

## Materials and methods

### Zebrafish husbandry

Wild-type zebrafish (*Danio rerio*, AB/TL strain) were maintained at 28 °C in a recirculating system with controlled water quality (pH 7.0–7.4, conductivity 500–600 µS/cm) under a 12/12-h light/dark cycle. Embryos were collected from natural spawning and raised at 28.5 °C in E3 medium (5 mM NaCl, 0.17 mM KCl, 0.33 mM CaCl_2_, 0.33 mM MgSO_4_) with regular monitoring for health and development. Zebrafish husbandry was conducted according to “The Zebrafish Book” (Kimmel et al., 1995; Westerfield, 2007). All key reagents and software tools are reported with corresponding Research Resource Identifiers (RRIDs) where available. All experimental procedures were performed in compliance with the Canadian Council on Animal Care guidelines and approved by the local ethics committee of the INRS (protocols 1605-01 and 2005-01).

### Experimental design

Based on preliminary toxicological assessments, appropriate doses of VPA and cocoa polyphenol-rich extract were established. The study involved four groups: control, cocoa-treated, VPA-treated, and VPA + cocoa co-treated zebrafish. Embryos were exposed to VPA, alone or in combination with cocoa extract, from 6 h post-fertilization (hpf) to 4.5 days post-fertilization (dpf). Cocoa extract treatment was administered from 6 hpf to 6 dpf, alone or in combination with VPA. Each group consisted of six independent batches, with each containing 20 larvae. Three batches from each group were processed for haematoxylin and eosin (H&E) staining. For the remaining three batches, behavioral analysis was conducted using the DanioVision system (Noldus, Leesburg, VA, USA), a high-throughput platform for monitoring zebrafish larvae. Following this analysis, batches were flash-frozen and stored at − 80 °C for subsequent metabolomic and proteomic analyses. All experiments were conducted at 6 dpf, prior to gonadal differentiation; at this stage, sex cannot be phenotypically or genotypically distinguished, and larvae are therefore considered sexually undifferentiated (von Hofsten & Olsson, 2005).

### VPA model validation

VPA was purchased from Sigma-Aldrich (Etobicoke, Ontario). To establish an appropriate concentration of VPA for inducing an ASD-like phenotype in zebrafish, an initial toxicological screening was conducted using a range of concentrations (5 to 500 µM), based on previous studies (Chen et al., 2018; Lee et al., 2018; Dwivedi et al., 2019; Messina et al., 2020; Brotzmann et al., 2021; Robea et al., 2021). Embryos were exposed to VPA in E3 medium, freshly prepared and replaced daily, from 6 hpf until 4.5 dpf, at which point they were switched to E3 only for the duration of the experiment (until 6 dpf), due to high toxicity observed after 4.5 dpf of exposure.

Behavioral assessments were conducted using DanioVision system with parameters set to detect hypoactivity, hyperactivity, and other ASD-like phenotypes, as described below. Gross morphology and survival were assessed. The larvae were observed under a Leica MZ16 FA stereomicroscope equipped with a DFC7000 T digital camera (Leica Microsystems, Rijswijk, The Netherlands). Survival was recorded daily, and morphological phenotypes such as pericardial edema, head malformations, and axial curvature were documented. The concentration of VPA was considered excessive if larvae did not survive up to 6 dpf or displayed severe toxicity (e.g., paralysis). Concentrations inducing no visible morphological or behavioral differences compared to controls were deemed too low to model ASD phenotypes effectively.

### Toxicology of cocoa polyphenol-rich extract

Cocoa powder was sourced from CocoaVia™ (Mars Canada Ltd., Bolton, Ontario). Concentrations for cocoa powder refer to the concentration of (−)-epicatechin present according to the CocoaVia™ nutrition facts label. To determine a safe concentration of cocoa polyphenols, a range of (−)-epicatechin doses ranging from 2.5 to 50 µM was tested and their effects on larval survival and hatching were assessed. To ensure consistent dosing, cocoa powder was weighed using an analytical balance (accuracy ± 0.01 mg) and dissolved in E3 medium. The cocoa-E3 solution was centrifuged at 10,000 x g for 5 min, and the supernatant was used for zebrafish treatment. Embryos were treated with cocoa powder from 6 hpf until 6 dpf. The medium was prepared freshly and replaced daily to maintain the final concentration of treatment.

### Behavioral analysis

Larval zebrafish at 6 dpf were individually placed into single wells of a 96-well plate containing 250 µL of E3 medium. The plates were then transferred to the DanioVision^®^ recording chamber (Noldus) and allowed to habituate for 1 h before the start of the experiment. Locomotor activity was assessed as an early and robust neurobehavioral endpoint in larval zebrafish. More complex ASD-related behaviors such as social preference and shoaling are typically evaluated at later developmental stages (> 10–14 dpf) involving more mature neural circuitry. Larval locomotor activity was monitored using EthoVision XT12 software (Noldus, RRID: SCR_000441), which automatically tracks and quantifies the movement of zebrafish larvae. The software was configured to measure total swimming distance, mean velocity, and activity burst patterns across light-dark cycles. Data were collected at a sampling rate of 30 frames per sec, with the experiment spanning several hours to capture diurnal behavioral rhythms.

### Gut morphology assessment

Gross gut morphology of zebrafish larvae at 6 dpf was assessed using hematoxylin and eosin (H&E) staining. Larvae were fixed in paraffin, and sections were stained with haematoxylin (StatLab) for 4 min, washed with alcohol-acid, and rinsed with tap water. Sections were then soaked in saturated lithium carbonate solution for 10 s and then rinsed with tap water. Finally, staining was performed with Eosin Y (StatLab) for 2 min, and sections were mounted under a coverslip with Permount mounting media (Fisher Scientific, Saint-Laurent, Quebec). The following sections were used to examine the gut: sagittal, transverse, and coronal planes. The gastrointestinal structure was noted as well as the placement of organs surrounding the gut such as the liver. All images were acquired at 260× magnification using a Zeiss Axio Zoom stereomicroscope (Zeiss, Germany) equipped with an iPhone 6 camera.

### Metabolomics

#### Sample preparation

Zebrafish larvae (20 per sample) were homogenized in 500 µL of cold methanol using a ultrasonic probe (Branson Digital Sonifier, Emerson Electric, St. Louis, MO) for 2 × 10 s on ice. Samples were then centrifuged at 14,000 rpm for 8 min at 4 °C using a 5424R centrifuge (Eppendorf, Hamburg, Germany). The supernatant was separated for untargeted metabolomics, while the protein pellet was processed for proteomics. The supernatant was dried under a nitrogen stream and reconstituted in 200 µL of 25% methanol for metabolomics analysis.

#### LC-MS/MS for untargeted metabolomics

Untargeted metabolomics was performed using a Shimadzu Nexera UHPLC system coupled with a high-resolution tandem mass spectrometer (Sciex TripleTOF 5600+). Detailed chromatographic gradients and MS acquisition parameters are provided in Supplementary Methods. Only features detected in all samples of at least one group were retained for further analysis, and all samples were injected within one batch. Metabolomics data was normalized using the total sum of areas for each sample.

### Proteomics

#### Sample preparation

Protein pellets were resuspended in 50 µL of 7 M urea/2 M thiourea and sonicated in a water bath for 15 min. 200 µL of ammonium bicarbonate (ABC) buffer (100 mM, pH 8.5) was added, and the sample was sonicated for 3 × 5 s on ice. Samples were centrifuged (14,000 rpm, 2 min), and protein concentrations were determined using the Bradford assay (Bio-Rad). A total of 50 µg protein (topped up to 400 µl ABC buffer) was reduced with 8 µL of 100 mM DTT at 37 °C for 15 min, followed by alkylation with 12 µL of 100 mM iodoacetamide at 37 °C for 30 min in the dark. Proteins were digested with 2 µg trypsin (Sigma-Aldrich) at 37 °C for 4 h with gentle agitation. After digestion, peptide digests were desalted on Oasis HLB cartridges (Waters, 1 cc 30 mg) and dried in a SpeedVac concentrator (Thermo Fisher Scientific). Peptides were reconstituted in 120 µL of 10% acetonitrile (ACN) + 0.2% FA for subsequent analysis.

#### LC-MS/MS for proteomics

Proteomics analysis was performed using the same system as for metabolomics analysis. Detailed chromatographic gradients and MS acquisition parameters are provided in Supplementary Methods. Data processing was carried out using ProteinPilot 5.0 software (Sciex, RRID: SCR_018681) to generate peptide and protein identification lists, which were used to create an ion library for quantification using SWATH (Sciex OneOmics). Criteria for protein identification included a 1% local false discovery rate (FDR) and quantification based on up to four peptides per protein, and three transitions per peptide.

#### Omics data analysis

Data analysis was performed using R (version 4.3.2). Natural log transformation was applied to normalize metabolomics and proteomics datasets. Intensities of features with similar identifications were averaged. The homogeneity of variance across groups was confirmed using Levene’s Test. Analysis of variance (ANOVA) was applied feature-wise to detect differences between treatment groups, followed by Benjamini–Hochberg FDR correction at a threshold of 20%, given the exploratory nature of the work. Significant features were further examined using Tukey’s HSD test. Metabolites and proteins were considered significantly different at *p*-value ≤ 0.05 and trending toward significance at *p*-value > 0.05 and < 0.1 (based on Tukey’s HSD test). Fold changes were calculated using raw (non-log-transformed) data as the ratio of mean metabolite or protein intensities between treatment groups. Correlation analysis using Pearson’s correlation was performed to examine relationships between proteins and metabolites that were significant or trending toward significance across all pairwise group comparisons. Normality of the data was assessed prior to analysis, and correlation *p*-values were adjusted using Benjamini–Hochberg correction.

Principal component analysis (PCA) was applied to visualize clustering patterns among samples, using MixOmics (v6.25.1, RRID: SCR_016889) R package. Prior to PCA, datasets were Pareto-scaled. Pathway enrichment analyses were based exclusively on univariate statistical results. Joint pathway analysis integrating metabolomics and proteomics results was performed using MetaboAnalyst 6.0 (RRID: SCR_015539). Enrichment analysis was based on hypergeometric test with degree centrality as the pathway topology measure. The input lists included metabolites and proteins that were significant or showed a trend toward significance between the compared groups, given the exploratory nature of the study. FELLA pathway analysis was also applied using FELLA R package. FELLA uses a hierarchical KEGG-based knowledge graph and propagates signals from input metabolites to identify enriched pathways and potential crosstalk within the network (Picart-Armada et al., 2018). The network graph was built using diffusion matrices (matrices = “diffusion”), a normality approximation for scoring (normality = “diffusion”), and 50 iterations. The input list included significant metabolites and those trending toward significance between the compared groups.

### Statistical analysis

All zebrafish experiments were performed in triplicate (*N* = 3 biological replicates), and each biological replicate consisted of 20 larvae (*n* = 20), providing biological robustness while maintaining feasibility for exploratory omics analyses. Behavioral and gut morphology analyses were also conducted in triplicate, with at least 12 larvae per batch (*n* = 12). This sample size was selected based on prior experience with similar behavioral and imaging experiments. Data are presented as Mean ± SEM. For non-omics data, statistical significance was set at *p*-value < 0.05. Differences in survival proportions across treatment groups were assessed using 95% confidence intervals with Bonferroni correction. Behavioral endpoints, including total distance moved, as well as gut villus length measurements across treatment groups, were analyzed using one-way ANOVA followed by Tukey’s HSD post hoc test.

## Results

### VPA model validation

VPA concentrations exceeding 10 µM significantly reduced larval survival, as shown in Supplementary (Fig. S1). Zebrafish treated with concentrations above 25 µM exhibited mortality within 24 h of exposure. Therefore, a narrower concentration range (1, 3, 5, and 10 µM) was tested to identify a suitable model for ASD. Survival, hatching, and deformity rates over time are illustrated in Fig. S2. Notably, both 5 and 10 µM VPA had significantly reduced survival with severe morphological deformities, such as pericardial edema.

Behavioral analysis (Fig. S3) indicated that zebrafish exposed 10 µM VPA exhibited no significant difference in total swimming distance relative to the control group (*p*-value = 0.8). Due to the high incidence of morphological deformities and poor survival at 5–10 µM concentrations, only 1 and 3 µM were deemed viable for further experimentation. Ultimately, 3 µM VPA was selected as the optimal concentration for inducing anxiety-like behavior in zebrafish while ensuring sufficient survival to 6 dpf. This dose was preferred over 1 µM to align with previous studies employing 3 µM VPA, thereby supporting the development of a robust and reproducible zebrafish model of ASD.

### Toxicology of cocoa polyphenol-rich extract

Concentrations above 2.5 µM (−)-epicatechin significantly reduced larval survival compared to control and 2.5 µM groups (Fig. S4a). Additionally, concentrations above 5 *µ*M delayed embryo hatching with markedly lower hatching success compared to control and lower-dose groups (Fig. S4b). While control, 2.5 and 5 *µ*M treatments had a 100% hatch rate, higher concentrations resulted in substantially lower hatching success. Therefore, 2.5 *µ*M (−)-epicatechin was selected for the subsequent experiments, as it maintained normal hatching and survival rates without inducing toxicity.

### Effect of cocoa treatment

#### Survival

Survival analysis showed that zebrafish treated with cocoa had survival rates comparable to the control group, indicating no adverse effects from the treatment (Fig. S5). Notably, the VPA + cocoa group of zebrafish exhibited slightly improved survival rates than the VPA-alone group, suggesting a potential protective effect of the cocoa against VPA-induced toxicity.

#### Behavioral functionality

**Fig. 1:**
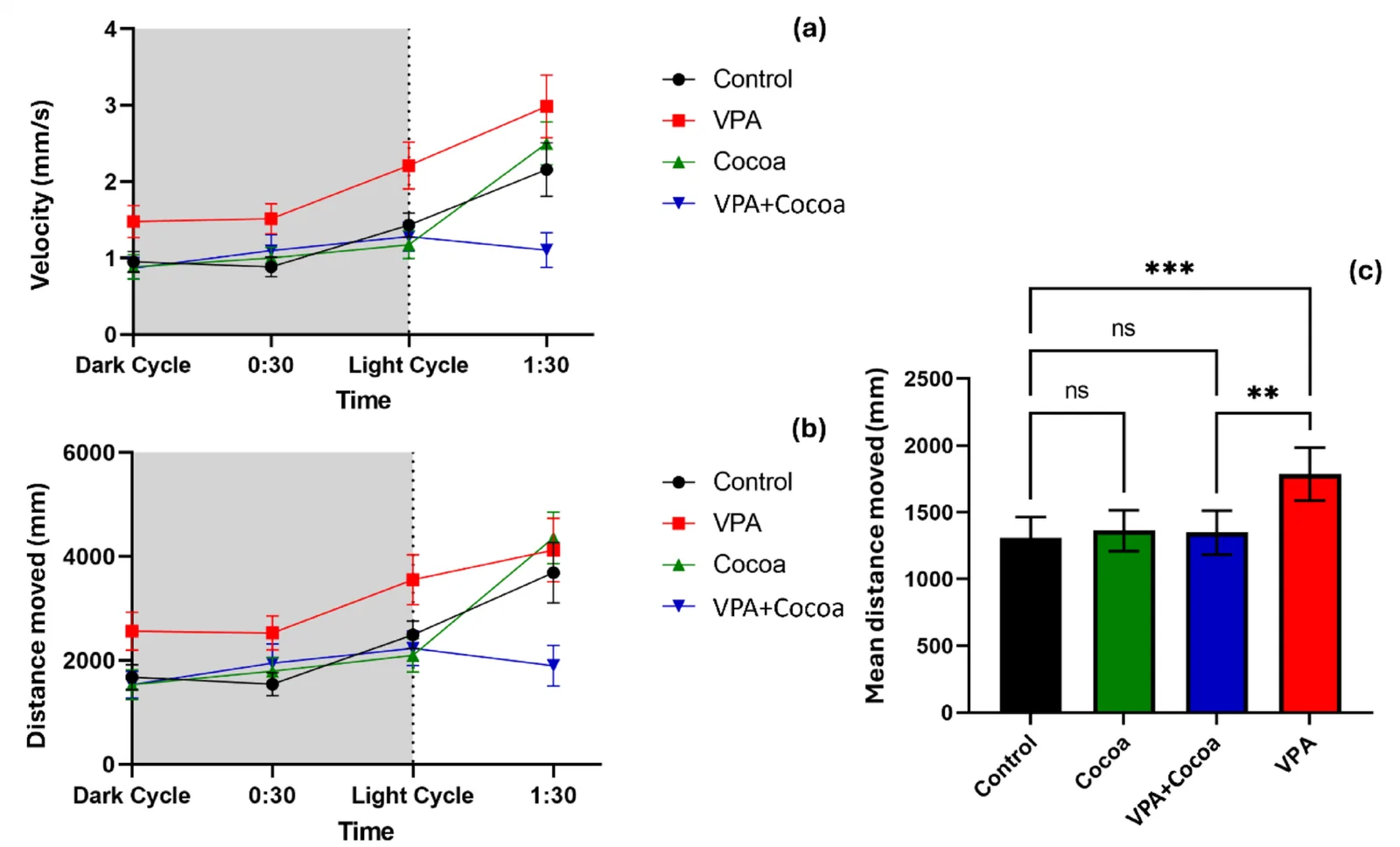
Behavioral analysis of zebrafish larvae following VPA and cocoa treatments. **a**, **b** Locomotor activity of control, VPA, Cocoa and VPA+Cocoa treatments showing velocity (**a**) and distance swum (**b**) in response to stress-inducing change in environment (dark to light). The shaded area indicates the dark cycle, and the transition to the light cycle is marked by the dashed line. **c** Mean total distance moved compared across groups (*N* = 3, *n* = 12). Data are presented as mean ± SEM. Statistical significance is indicated as ns (not significant), ** (*p* < 0.01), and *** (*p* < 0.001)

Behavioral assays conducted using DanioVision (Fig. 1) revealed that VPA-treated zebrafish displayed anxious-like behavior, characterized by increased swimming velocity and distance during both light and dark cycles of the assay, compared to the control, cocoa-only and VPA + cocoa groups. In contrast, VPA + cocoa group displayed calm behavior similar to controls during the dark cycle and maintained this behavioral profile after transitioning to the light phase. Their swimming patterns closely resembled those of the control and cocoa-only groups, with significantly reduced total swimming distances compared to the VPA-alone group.

#### Gut morphology

**Fig. 2:**
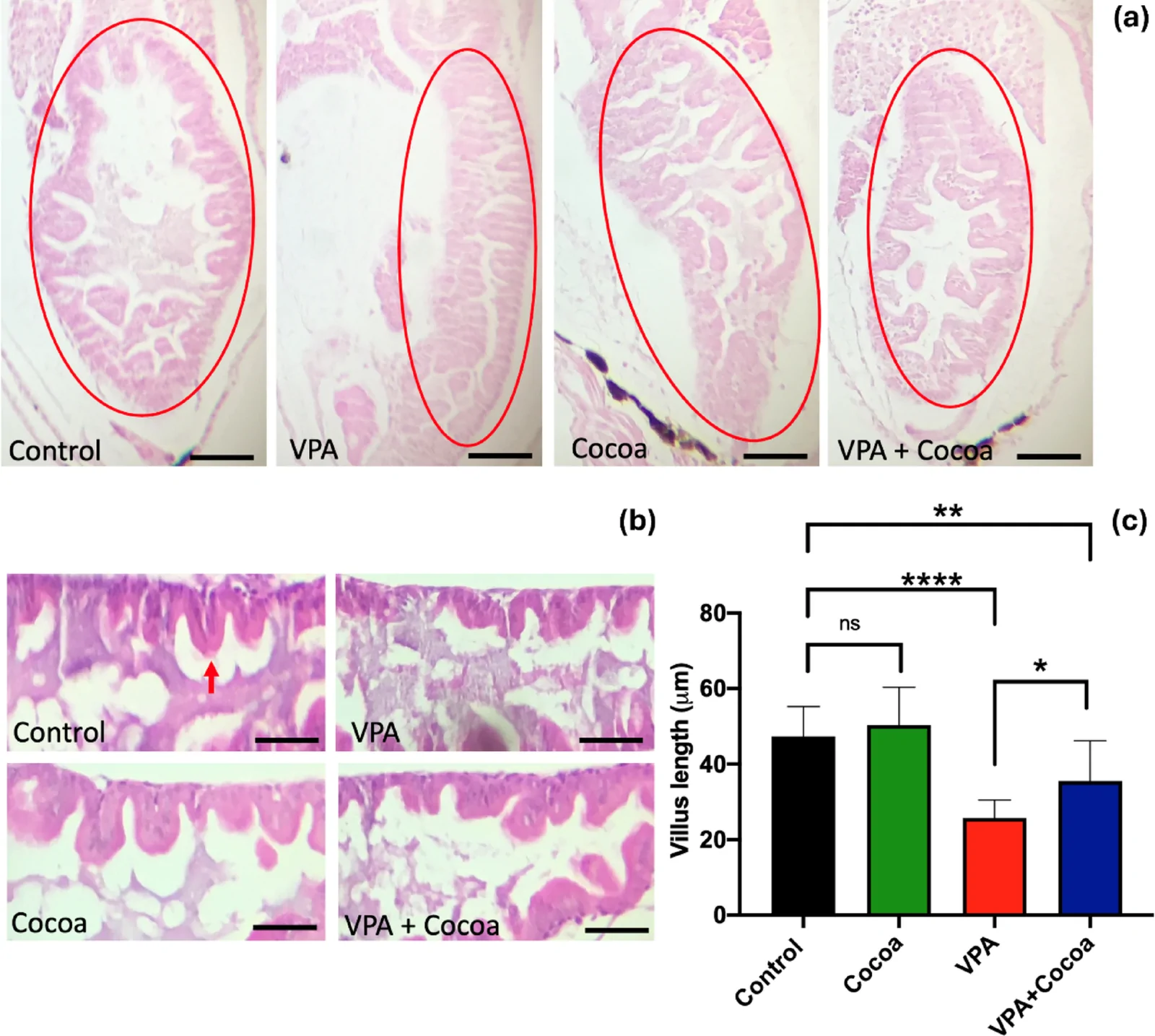
Hematoxylin and eosin (H&E) staining of zebrafish larvae at 6 days post-fertilization. **a** Representative coronal sections from control, VPA-treated, cocoa-treated, and VPA + cocoa co-treated groups are shown. The intestinal region is highlighted (red circles) to facilitate comparison of intestinal architecture across conditions. All images were acquired under identical imaging conditions. **b** Representative intestinal sections showing villus length (red arrow) in the different groups. **c** Comparison of mean villus length across treatment groups (*N* = 3, *n* = 12). Scale bar = 50 μm. Data are presented as mean ± SEM. Statistical significance is indicated as ns (not significant), * (*p* < 0.05),** (*p* < 0.01) and **** (*p* < 0.0001)

Histological examination of zebrafish gut morphology (Fig. 2A, B) further supported the protective effects of cocoa powder. Control zebrafish displayed normal intestinal structure with well-defined folds. In contrast, VPA-treated zebrafish exhibited pronounced structural abnormalities, including deformed gut tissue with less folds and significantly shorter villi compared to the control group. Notably, co-treatment of VPA-treated zebrafish with cocoa powder maintained gut morphology to a state resembling that of the control group, characterized by well-rounded and properly organized intestinal structures. Mean villus length was significantly greater in the VPA + cocoa group compared to the VPA-alone group. These findings suggest that cocoa treatment played a role in protecting gut integrity in VPA-treated zebrafish.

#### Metabolomics and proteomics

**Fig. 3:**
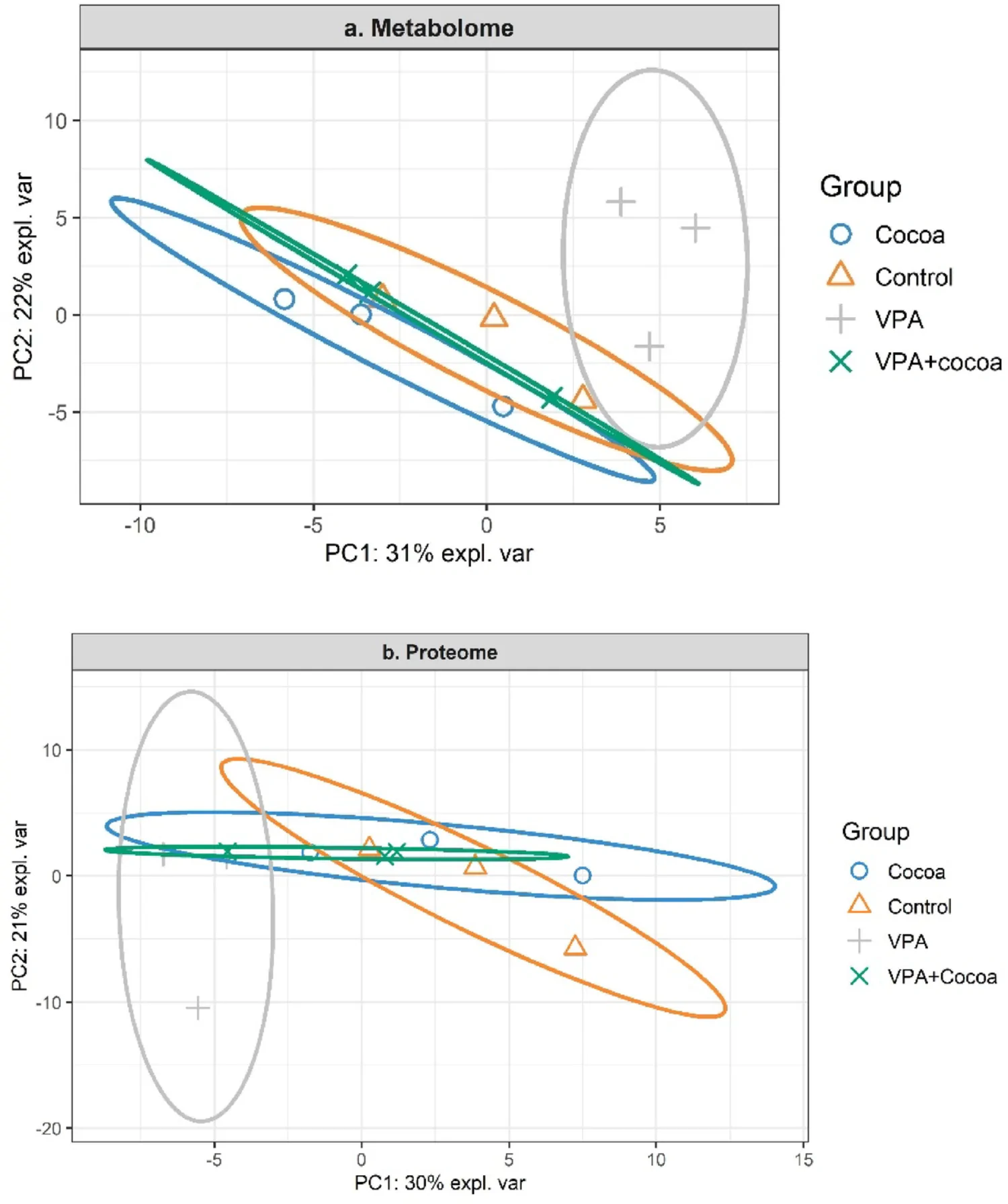
PCA scores plots of **a** metabolomic and **b** proteomic profiles. Each point represents an individual sample, and ellipses represent the 95% confidence intervals for each group. The percentage of variance explained by each principal component is indicated on the axes (PC1 and PC2). Separation and overlap among groups reflect differences and similarities in metabolomic and proteomic profiles across experimental conditions

Metabolomic and proteomic analyses identified 256 metabolites and 203 proteins, respectively. PCA visualization of both datasets revealed distinct clustering among treatment groups (Fig. 3). The VPA-treated zebrafish group formed a distinctly separate cluster, indicating a substantial shift in both metabolomic and proteomic profiles following VPA exposure. In contrast, zebrafish co-treated with VPA and cocoa clustered more closely with the control and cocoa-treated groups, indicating that cocoa partially mitigated the perturbations induced by VPA.

**Fig. 4:**
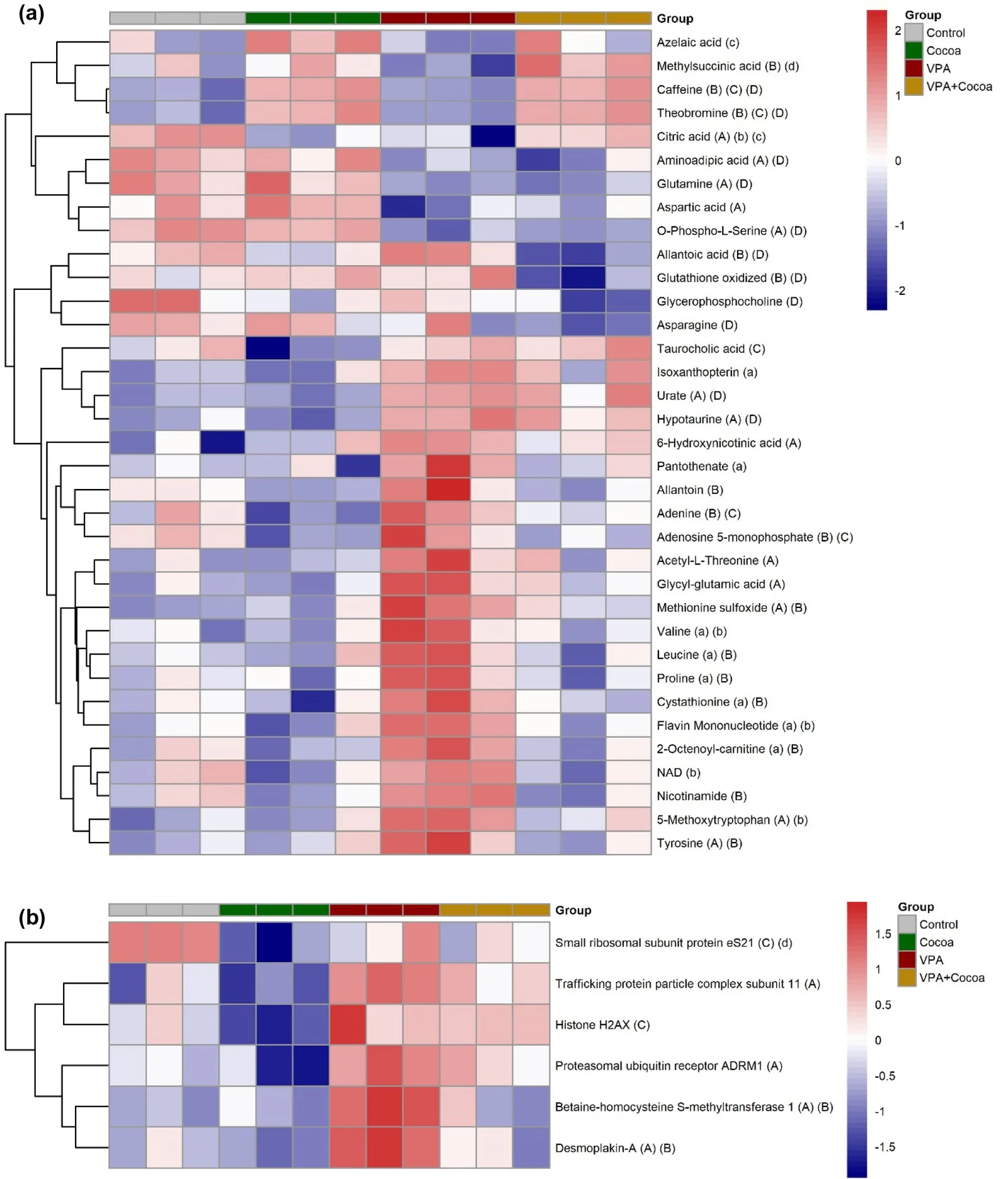
Intensity heatmap of altered metabolites (**a**) and proteins (**b**) across treatment groups. Color intensity represents scaled abundance levels (red, higher; blue, lower) across samples. Columns correspond to individual samples, and rows represent metabolites or proteins. Hierarchical clustering is shown for features. Letters denote significant (capitals, A-D) and trend toward significant differences (lowercase, a-d) between: (A/a) VPA vs. Control, (B/b) VPA + Cocoa vs. VPA, (C/c) Cocoa vs. Control, (D/d) VPA + Cocoa vs. Control. For proteins, UniprotKB accession numbers are provided in parentheses as follows: Betaine-homocysteine S-methyltransferase 1 (Q32LQ4); Desmoplakin-A (A0A8M2BID5); Histone H2AX (Q7ZUY3); Proteasomal ubiquitin receptor ADRM1 (Q6NZ09); Small ribosomal subunit protein eS21 (Q7ZUG5); Trafficking protein particle complex subunit 11 (Q1RLX4)

To further investigate metabolic and proteomic alterations, univariate analysis was performed, revealing that VPA treatment significantly increased the levels of multiple metabolites, including methionine sulfoxide, urate, hypotaurine, 6-hydroxynicotinic acid, 5-methoxytryptophan, glycyl-glutamic acid, and tyrosine, while reducing O-phospho-L-serine, glutamine, and citric acid, among other metabolites, compared to controls (Table S1). Trends toward increases in cystathionine, valine, leucine, proline, pantothenate, isoxanthopterin, flavin mononucleotide, and 2-octenoyl-carnitine were also observed (*p* ≤ 0.1). Differential metabolite intensities across groups are shown in Fig. 4a.

Interestingly, cocoa co-treatment mitigated many of these alterations. Compared with VPA alone, the VPA + cocoa group showed significantly lower levels of methionine sulfoxide, cystathionine, tyrosine, proline, leucine, and 2-octenoyl-carnitine, with trends toward reductions in 5-methoxytryptophan, flavin mononucleotide, and valine. Citric acid tended to be higher with cocoa co-treatment. In addition, cocoa co-treatment significantly reduced oxidized glutathione, ophthalmic acid, allantoic acid, allantoin, nicotinamide, adenine, and adenosine monophosphate, and tended to reduce nicotinamide adenine dinucleotide (NAD^+^) compared with VPA-alone group. Cocoa-derived metabolites, specifically caffeine and theobromine, were significantly higher in both cocoa-alone and VPA + cocoa groups compared to controls and VPA alone, confirming the bioavailability of cocoa-derived compounds in the zebrafish larvae.

Univariate proteomic analysis (Fig. 4b, Table S1) revealed several proteins were significantly altered by VPA. Compared to controls, VPA led to increased levels of trafficking protein particle complex subunit 11 (TRAPPC11), proteasomal ubiquitin receptor (ADRM1), betaine-homocysteine S-methyltransferase 1 (BHMT-1), and desmoplakin-A. Cocoa co-treatment mitigated these effects as it reduced the levels of BHMT-1 and desmoplakin-A relative to VPA alone. In addition, cocoa-alone treatment significantly reduced the abundance of histone H2AX and small ribosomal subunit protein eS21 compared to controls.

**Fig. 5:**
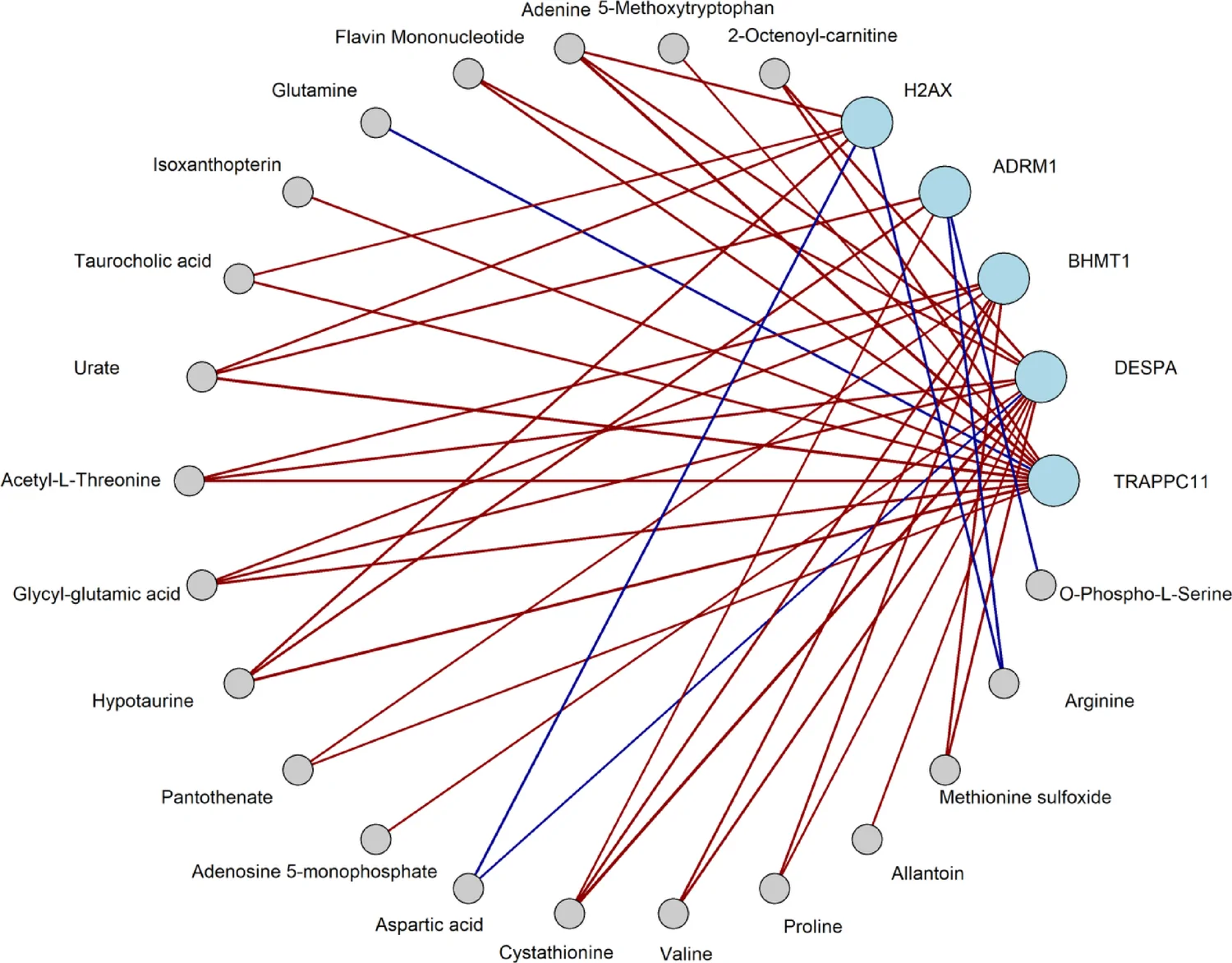
Bipartite protein-metabolite correlation network. Bipartite network showing Pearson correlations between altered proteins and metabolites across all pairwise group comparisons. Only significant correlations are shown (adjusted *p*-value < 0.05). Blue and gray nodes denote proteins and metabolites, respectively, whereas red and blue edges represent positive and negative correlations. Edge thickness reflects the absolute Pearson correlation coefficient (|r|), which ranged from 0.72 to 0.89. BHMT1: Betaine-homocysteine S-methyltransferase 1; DESPA: Desmoplakin-A; H2AX: Histone H2A variant X; ADRM1: Proteasomal ubiquitin receptor; TRAPPC11: Trafficking protein particle complex subunit 11

Altered proteins, across all pairwise comparisons, showed significant correlations to several metabolites, as shown in the correlation network (Fig. 5). Among the proteins, DESPA and TRAPPC11 exhibited the largest number of significant correlations, most of which were positive. Metabolites linked to sulfur amino acid and redox metabolism, including cystathionine, hypotaurine, and methionine sulfoxide, showed correlations with multiple proteins, particularly BHMT1, DESPA, and TRAPPC11. Small ribosomal subunit protein (eS21) did not correlate significantly to any of the altered metabolites (adjusted *p*-value > 0.05).

Joint pathway enrichment analysis comparing VPA-treated zebrafish to controls revealed disruptions in aminoacyl-tRNA biosynthesis, pantothenate and CoA biosynthesis, and several amino acid pathways, including valine, leucine and isoleucine biosynthesis; alanine, aspartate and glutamate metabolism; cysteine and methionine metabolism; and arginine biosynthesis, all of which were significant at an FDR level of 20% (Fig. S6a). Cocoa co-treatment, in comparison with VPA, was associated with enrichment of the aminoacyl-tRNA biosynthesis and valine, leucine and isoleucine biosynthesis, cysteine at an FDR level of 20% (Fig. S6b).

**Fig. 6:**
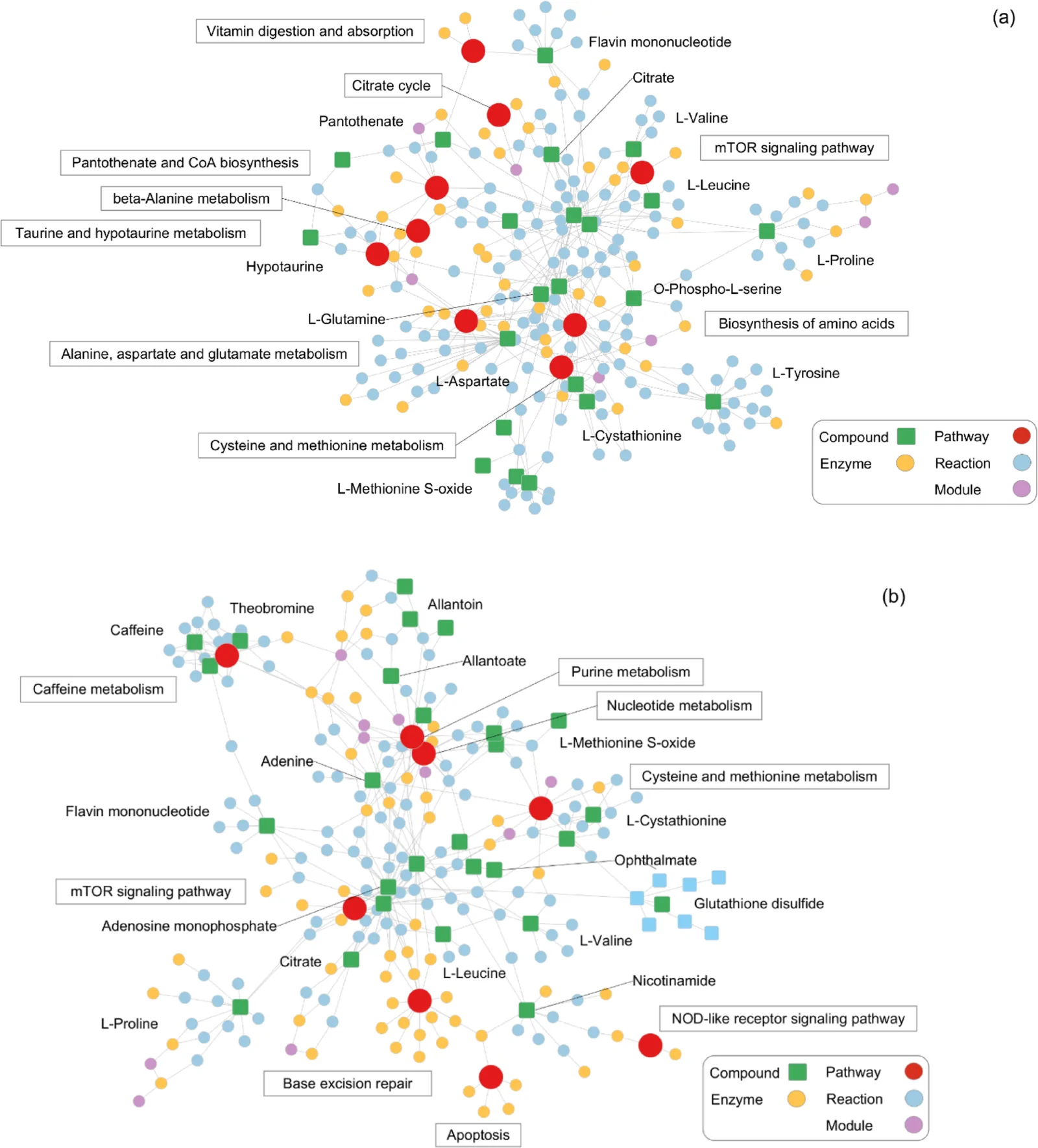
FELLA-based pathway enrichment network analysis. **a** VPA compared to control group. **b** VPA + cocoa compared to VPA group. Networks display significantly enriched pathways and their associated metabolites mapped onto the KEGG-based interaction network. Nodes represent biological entities, including metabolites (compounds), enzymes, reactions, modules, and pathways, as indicated in the legend, and edges represent their relationships within the network. Labeled nodes correspond to enriched pathways and input metabolites contributing to the enrichment. Only pathways with p-score < 0.05 are shown.

FELLA pathway analysis identified several significantly enriched pathways in response to VPA treatment (VPA vs. control), including citrate cycle, mTOR signaling, cysteine and methionine metabolism, pantothenate and CoA biosynthesis, and taurine and hypotaurine metabolism, among others. Comparison of the VPA + cocoa group to VPA alone revealed enrichment of mTOR signaling, cysteine and methionine metabolism, nucleotide and purine metabolism, caffeine metabolism, and apoptosis, among other pathways. Network visualization of these enriched pathways is presented in Fig. 6.

## Discussion

This study validates the VPA-induced zebrafish model of ASD and supports cocoa polyphenol-rich extract as a promising therapeutic candidate. While no ideal VPA-induced zebrafish model of ASD has yet been established, recent studies continue to support its utility for modeling ASD-like behavioural phenotypes (Camussi et al., 2025). Previous studies have shown that low-dose VPA exposure at 1 µM during early developmental stages can induce behavioral alterations, including social deficits and impaired locomotor activity (Karimi et al., 2023; Geng et al., 2023). In line with these findings, we observed significant alterations in locomotor activity at 1 µM VPA, supporting the sensitivity of this model to subtle neurobehavioral disruptions.

Toxicological assessment, in our study, showed that VPA exposure produced teratogenic effects at concentrations exceeding 5 µM, with pronounced lethality and morphological abnormalities at higher concentrations (15–25 µM), which is consistent with previous research (Karimi et al., 2023), reinforcing the dose-dependent toxicity profile of VPA in developing zebrafish. These effects are largely attributed to VPA’s activity as a histone deacetylase inhibitor, leading to epigenetic dysregulation during critical windows of neurodevelopment (Gurvich et al., 2005; Messina et al., 2020; Muhsen et al., 2021). In contrast, polyphenol-rich cocoa extract, at a physiologically relevant concentration of 2.5 µM of (−)-epicatechin, was non-toxic and preserved gut architecture, which VPA severely disrupted. Gut histological observations, both qualitative and quantitative, including villus length measurements, were consistent with a protective effect of cocoa extract against VPA-induced disruption. While these findings support a beneficial effect of cocoa cotreatment on gut integrity, future studies are warranted to further evaluate additional quantitative indices, such as epithelial thickness and permeability markers.

Behaviorally, zebrafish co-treated with VPA and cocoa exhibited normalized swimming patterns comparable to controls during the dark phase of motor activity assessment using the DanioVision system. Notably, co-treated larvae also showed enhanced behavioral stability under light-induced stress, in contrast to the anxiety-like responses observed in controls. This behavioral rescue is consistent with the reported effects of cocoa bioactive compounds in promoting brain-derived neurotrophic factor (BDNF) signaling, a key regulator of synaptic plasticity, and in restoring excitatory-inhibitory synaptic balance, both of which are disrupted by VPA (Cimini et al., 2013; Socci et al., 2017; Qi et al., 2022). While locomotor alterations are an early neurobehavioral phenotype in zebrafish ASD models, core ASD behaviors such as social interaction and shoaling emerge at later developmental stages that should be examined in future studies for a more comprehensive behavioral assessment.

Metabolomic profiling revealed VPA-induced disruptions in sulfur amino acid metabolism. Cysteine and methionine pathways are increasingly implicated in ASD pathogenesis (Indika et al., 2021), and they integrate amino acid turnover with methylation and redox homeostasis (Lu, 2000; Indika et al., 2021). Consistent with ASD-associated metabolic disturbances, characterized by reduced DNA methylation and elevated oxidative distress (Thorsen, 2020), VPA upregulated BHMT-1, an enzyme that remethylates homocysteine to methionine and supports the synthesis of S-adenosylmethionine (SAM), the primary methyl donor for DNA and protein methylation. This upregulation likely represents a compensatory response to maintain SAM-dependent methylation under VPA-induced stress (Sternbach et al., 2021).

Our findings contrast with reports of reduced hepatic BHMT expression in mice prenatally exposed to VPA (Huang et al., 2019), as well as reduced BHMT expression in muscle tissue of VPA-exposed chicken embryos (Chen et al., 2014). Another study reported no significant effect of acute VPA exposure on hepatic BHMT activity in adult rats (Ubeda et al., 2002). Several factors may contribute to these discrepancies, including differences in species, developmental stage, VPA dosage, and duration of exposure, all of which are likely to differentially influence methylation-related compensatory responses. Nevertheless, our findings further support the involvement of one-carbon metabolism disturbances in ASD-related pathophysiology (Ornoy et al., 2019).

VPA reduced O-phospho-L-serine, a precursor for cysteine and phosphatidylserine biosynthesis. Phosphatidylserine supports membrane integrity, synaptic signaling, and neuroinflammatory regulation (Ma et al., 2022) and its depletion parallels reports of reduced blood levels in ASD (El-Ansary et al., 2011). O‑phospho‑L‑serine is also the immediate substrate of phosphoserine phosphatase in the phosphorylated serine pathway, and its observed decrease under VPA exposure indicates reduced substrate availability for the final step of serine biosynthesis. Lower serine availability restricts entry into the trans‑sulfuration pathway, in which serine combines with homocysteine to form cystathionine and is subsequently converted to cysteine (Indika et al., 2021; Lu, 2020). Although a bottleneck at O‑phospho‑L‑serine predicts constrained serine‑dependent flux, we noted a trend toward higher cystathionine with VPA. This pattern is coherent under oxidative distress conditions whereby cystathionine β‑synthase is up‑regulated leading to the shunting of homocysteine toward cystathionine as a protective sink despite reduced serine input (Romero et al., 2015). Moreover, the conversion of cystathionine to cysteine via cystathionine γ‑lyase can be rate‑limited by redox constraints, leading to cystathionine accumulation without efficiently replenishing cysteine pools (Niu et al., 2018). This is consistent with the heightened redox stress markers in the VPA group, including increased methionine sulfoxide. Together with mitochondrial stress markers, these data support cystathionine β‑synthase‑driven cystathionine build‑up coupled to impaired conversion to cysteine, ultimately limiting glutathione synthesis (Indika et al., 2021; Lu, 2020; Thorsen, 2020).

Other indicators of disrupted methylation and oxidative distress included a significant increase in methionine sulfoxide and hypotaurine in response to VPA. Formation of methionine sulfoxide, driven by oxidative distress, may deplete the pool of methionine required for SAM synthesis, thereby exacerbating methylation deficits (Lee, 2011). Consistently, the accumulation of hypotaurine may reflect enhanced oxidative catabolism of the reduced cysteine pool or impaired conversion to taurine (Stipanuk, 2011). Under conditions of redox stress and an altered NAD⁺/NADPH balance, the metabolic sequence in which cysteine is oxidized to cysteine sulfinic acid, then converted to hypotaurine, and ultimately to taurine may be disrupted, and the final oxidation step from hypotaurine to taurine could become inefficient, leading to the buildup of hypotaurine (Niu et al., 2018). This aligns with VPA-induced neurotoxicity, as taurine, a neuroprotective molecule, plays a critical role in neurogenesis, synaptogenesis, and excitatory/inhibitory balance (Rubio-Casillas et al., 2022). Alterations in hypotaurine and taurine metabolism have been reported in ASD, although findings are inconsistent (Kuwabara et al., 2013; Xu et al., 2021), which underscores the complexity of sulfur amino acid metabolism in ASD.

Tryptophan metabolism was profoundly affected, with a fourfold increase in 5-methoxytryptophan (5-MTP), indicating a diversion from serotonin production. This finding aligns with reports of reduced tryptophan metabolism and lower brain serotonin synthesis in ASD (Boccuto et al., 2013; Adamsen et al., 2014), despite paradoxical peripheral hyperserotonemia (Esposito et al., 2024), highlighting complex serotonergic dysregulation. The VPA-induced reduction in glutamine is likely due to inhibition of astrocytic glutamine synthetase (Zhang et al., 2024). Neuronal uptake of astrocyte-derived glutamine is critical for both glutamatergic (excitatory) and GABAergic (inhibitory) neurotransmission, so its reduction compromises excitatory–inhibitory balance (Badawy et al., 2020). Consistently, lower plasma glutamine has been observed in children with ASD and in VPA rodent models (Al-Otaish et al., 2018; Zieminska et al., 2018). Our findings also align with prior zebrafish metabolomic studies showing significant reductions in glutamine together with disruptions in the glutamatergic-GABAergic axis (Sun et al., 2025).

Furthermore, glycyl-glutamic acid, a dipeptide of glycine and glutamate, was elevated. Given its reported neurotrophic effects, this accumulation may represent a compensatory response to VPA-induced metabolic stress (Koelle et al., 1985). Tyrosine, a precursor for catecholamines, was also elevated. While tyrosine hydroxylase, the rate-limiting enzyme converting tyrosine to dopamine, is reportedly downregulated in VPA-treated animals (Cezar et al., 2018), dopamine concentrations show region-specific variability, with both increases and decreases observed (Kuo & Liu, 2022). Nonetheless, tyrosine elevation reflects perturbation of catecholaminergic pathways and underscores the broader impact of VPA on amino acid metabolism and neurotransmitter precursor pathways.

VPA-induced alterations strongly implicate mitochondrial dysfunction. VPA was reported to limit mitochondrial pyruvate uptake, reducing acetyl-CoA and tricarboxylic acid (TCA) cycle flux and impairing oxidative phosphorylation and ATP synthesis (Aires et al., 2008). Consistently, we observed decreased levels of citrate, the first intermediate of the TCA cycle, and aspartate, alongside a trending elevation in pantothenate, the precursor of coenzyme A. Notably, higher pantothenate levels in cord blood have been associated with a higher autism risk in children (Raghavan et al., 2023). Furthermore, VPA exposure increased 6-hydroxynicotinic acid, a catabolic oxidation product of nicotinic acid, a precursor in NAD⁺ biosynthesis. Accumulation of this catabolite along with the observed reduction of citrate and aspartate also suggests altered NAD⁺ redox balance, which is critical for oxidative phosphorylation (Takahashi et al., 2022; Yusri et al., 2025). This interpretation aligns with reported reduction in hepatic NAD⁺ levels in VPA-exposed rats (Moedas et al., 2016). Overall, our findings are consistent with ASD-associated deficits in energy metabolism (Frye, 2020).

Mitochondrial dysfunction is further supported by the trend toward elevated branched chain amino acids (BCAAs), particularly leucine and valine, since BCAAs are predominantly catabolized within the mitochondria (Gao et al., 2024). These findings align with reports linking BCAA accumulation to neurotransmitter imbalance, oxidative distress, and neuronal apoptosis (Cole et al., 2012; Fermo et al., 2023). Despite inconsistent findings, elevated BCAAs have been reported in children with ASD (Gao et al., 2024). VPA treatment appears to alter the mechanistic target of rapamycin (mTOR) signaling pathways. While the precise direction of alteration is difficult to ascertain, the observed metabolic pattern indicative of oxidative distress suggests suppression of mTOR complex 1 (mTORC1) activity (Wang et al., 2016). This interpretation aligns with findings of suppressed TORC1 signaling in postmortem fusiform gyrus tissue from individuals with idiopathic autism and VPA-exposed rats (Nicolini et al., 2015).

Interestingly, cocoa co-treatment mitigated several VPA-induced metabolic alterations, significantly reducing methionine sulfoxide, cystathionine, tyrosine, leucine, proline, and 2-octenoyl-carnitine, with trends toward lower 5-MTP, valine, and flavin mononucleotide. Cocoa also reduced oxidized glutathione and tended to increase citrate, suggesting improved redox homeostasis and mitochondrial function. Together, these findings indicate that cocoa polyphenols counteract VPA-induced disruptions in methylation, neurotransmitter balance, mitochondrial oxidative metabolism, and redox homeostasis.

Furthermore, cocoa reduced the levels of adenine, adenosine monophosphate (AMP), allantoin, and allantoic acid. AMP, a purine nucleotide derived from adenine, plays a central role in nucleotide interconversion and serves as a sensitive indicator of energy stress (Hardie, 2014). Allantoic acid, a downstream product of purine catabolism, reflects purine catabolic flux and oxidative burden (Cicero et al., 2023). The above observations imply that cocoa polyphenols modulate nucleotide homeostasis and purine catabolic flux, enhancing energy sufficiency and redox balance under VPA-induced stress. Our results align with a recent study on cocoa shell extract in rats, which reported improved purine metabolism and cellular repair mechanisms (Ramiro-Cortijo et al., 2025).

VPA exposure upregulated proteins implicated in neurodevelopment, including TRAPPC11, ADRM1, and desmoplakin-A. TRAPPC11, a component of cellular membrane trafficking machinery, is critical for clearing neurotoxic and misfolded proteins, processes to which neuronal cells are highly sensitive (Pawłowicz, 2023). Similarly, ADRM1 facilitates the degradation of misfolded proteins through the ubiquitin-proteasome system (Huang & Her, 2017). Disruptions in proteasomal function and vesicle trafficking machinery have been associated with neurodevelopmental disorders and intellectual disabilities (Huang & Her, 2017; Pawłowicz, 2023). Desmoplakin-A contributes to intercellular adhesion and regulates cell signaling, including neuronal activation and regulation of emotion-related behavior in mice (Otsubo et al., 2024). Importantly, cocoa co-treatment reduced the elevated levels of desmoplakin-A induced by VPA, suggesting restoration of structural integrity and intercellular signaling.

The correlation network provided further insights to the association patterns among altered proteins and metabolites. BHMT1 appeared to represent a primary putative hub within the network, exhibiting biologically coherent clustering with sulfur amino acid and redox-related metabolites, including cystathionine and methionine sulfoxide. H2AX appeared as a secondary hub showing correlations with metabolites linked to oxidative distress and mitochondrial dysfunction, consistent with the established role of H2AX in DNA damage signaling. In contrast, although DESPA and TRAPPC11 exhibited high connectivity, their correlations were diffuse and distributed across metabolically diverse pathways, suggesting broader cellular stress responses rather than pathway-specific clustering. ADRM1 showed more selective associations with metabolites linked to redox balance and amino acid metabolism, including hypotaurine, urate, cystathionine, arginine, and O-phospho-L-serine.

**Fig. 7:**
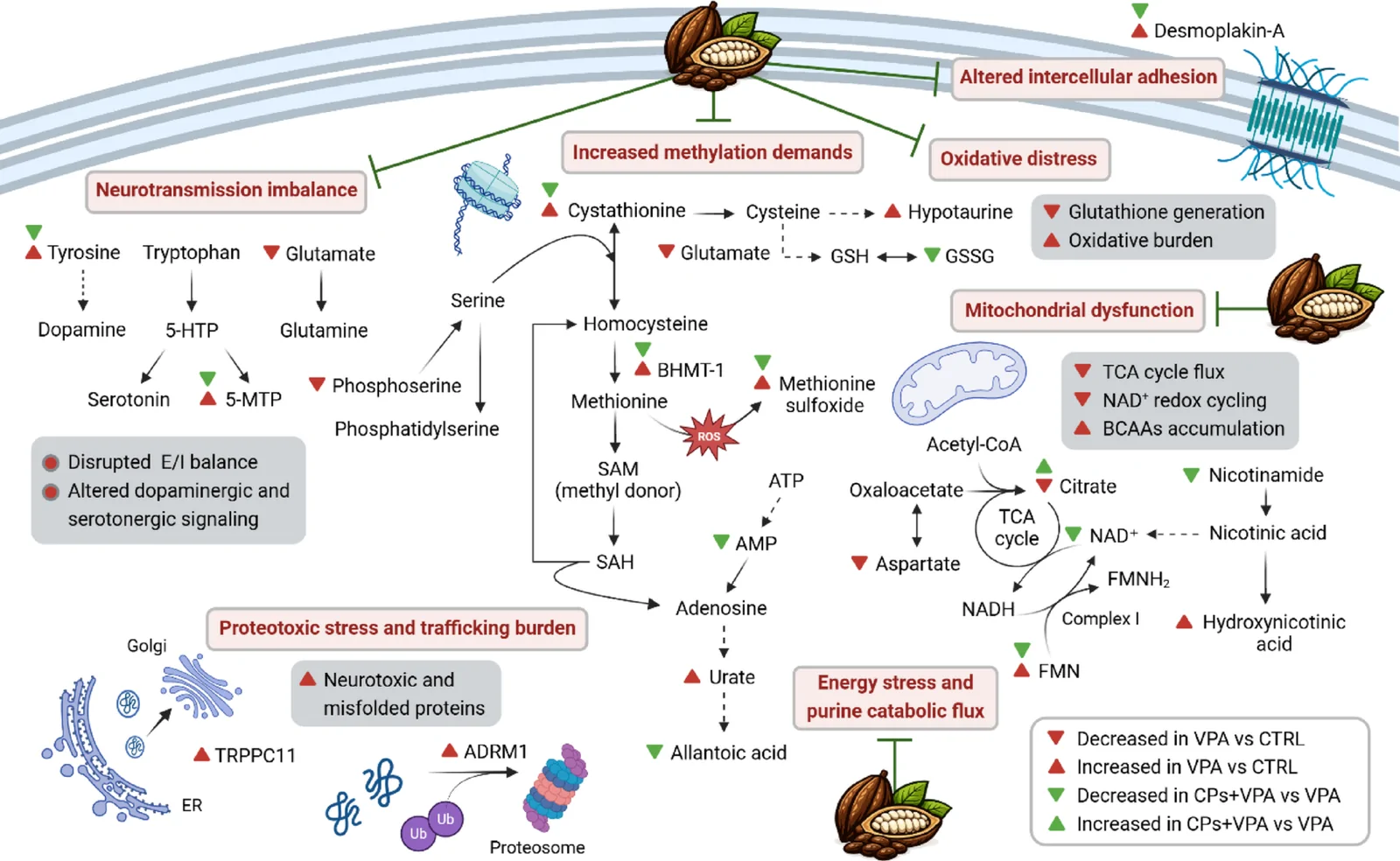
Integrated mechanistic overview of VPA‑induced metabolic and proteomic dysregulation and cocoa‑mediated mitigation. This schematic diagram summarizes the major metabolic pathways and protein networks perturbed by VPA exposure in zebrafish larvae and highlights the biochemical nodes where cocoa polyphenols exert mitigating effects. Metabolites and proteins shown were significantly altered or showed trends toward significance in the comparative analyses. Blunt-ended arrows denote inhibitory or mitigation effects. Dashed arrows indicate multi-step reactions. Red annotations and grey panels denote the inferred consequences of VPA‑induced perturbations, including increased methylation demands associated with sulfur amino acid metabolic perturbations and oxidative distress (elevated BHMT‑1, methionine sulfoxide, cystathionine, and hypotaurine with reduced phosphoserine); mitochondrial dysfunction (reduced citrate and aspartate with elevated FMN and BCAAs; leucine and valine); energy stress and purine catabolic flux (elevated urate), and neurotransmitter pathway dysregulation (altered tyrosine, tryptophan, and glutamine metabolism). VPA also upregulated proteins associated with cellular stress responses, including TRAPPC11, ADRM1, and desmoplakin‑A. Green annotations denote cocoa-mediated protection, including reductions in VPA-elevated metabolites (cystathionine, methionine sulfoxide, tyrosine, and 5-MTP), pathway-level mitigation of energy stress (reduced AMP and allantoic acid) and oxidative distress (reduced GSSG), and partial restoration of mitochondrial function (normalized citrate and FMN, and reduced nicotinamide and NAD^+^). Cocoa co‑treatment also reduced VPA‑induced elevations in BHMT‑1 and desmoplakin‑A, reflecting mitigation of methylation imbalance and cellular stress responses. Abbreviations: AMP, adenosine monophosphate; BCAAs, branched-chain amino acids; BHMT-1, betaine-homocysteine S-methyltransferase 1; CPs, cocoa polyphenols; CTRL, control; FMN, flavin mononucleotide; GSH, reduced glutathione; GSSG, oxidized glutathione; NAD⁺, nicotinamide adenine dinucleotide; SAH, S-adenosylhomocysteine; SAM, S-adenosylmethionine; 5-HTP, 5-hydroxytryptophan; 5-MTP, 5-methoxytryptophan

While metabolomic alterations observed in VPA-induced ASD models are not expected to fully recapitulate the metabolic heterogeneity of ASD patients, many affected pathways overlap with the established ASD-related biological frameworks. In the present study, VPA-induced oxidative distress was associated with disturbances in mitochondrial function and one-carbon metabolism, ultimately impairing neurotransmitter synthesis and neuronal signaling. These findings are consistent with oxidative, mitochondrial, and epigenetic abnormalities previously reported in ASD (Frye, 2020; LaSalle, 2013). Conversely, cocoa polyphenol treatment partially restored redox balance and metabolic homeostasis. An integrative mechanistic model summarizing VPA-induced metabolic and proteomic disturbances and sites of cocoa-mediated mitigation is shown in Fig. 7. Cocoa flavonoids, particularly flavanols such as epicatechin, are known to activate antioxidant pathways involving transcription factors such as nuclear factor erythroid 2-related factor 2 (Nrf2) (Martín & Ramos, 2016), modulate mitochondrial bioenergetics (Daussin et al., 2021) and influence one‑carbon metabolism and epigenetic regulation through effects on methyl donor pathways and DNA methylation dynamics (Pérez-Durán et al., 2026). Thus, there is a plausible mechanistic basis for the observed protective effects in which cocoa polyphenols act on multiple interconnected pathways.

Several limitations should be considered when interpreting the present findings. Experiments were conducted at the larval stage prior to sex differentiation; therefore, sex-specific effects could not be evaluated. Given the recognized sexual dimorphism in ASD prevalence and neurodevelopmental pathways, future studies using juvenile or adult zebrafish are warranted to assess potential sex-dependent responses to VPA exposure and cocoa polyphenol intervention. In addition, the limited sample size constrained statistical power. Accordingly, the present omics analyses should be interpreted as exploratory and hypothesis-generating, with emphasis placed on pathway-level alterations. Future larger-scale studies should focus on validating key pathways identified in this pilot analysis, utilizing qPCR, Western blotting and targeted metabolomics. Larger-scale investigations are required to validate these findings, identify the bioactive constituents underlying the observed effects, and assess their efficacy in additional translational models of neurodevelopmental dysfunction. Furthermore, although zebrafish provide a mechanistically informative model, limitations remain regarding higher-order neurobehavioral complexity and translational relevance to human ASD (Dreosti et al., 2020). Therefore, the present findings should be interpreted alongside future studies using complementary mammalian models.

In conclusion, VPA exposure generated a coordinated disturbance across mitochondrial metabolism, methylation pathways, redox homeostasis, neurotransmitter balance, and proteostasis, reflecting broad biochemical stress relevant to disturbed neuronal development and ASD pathophysiology. Notably, cocoa polyphenols partially restored these disrupted axes, normalizing key metabolic and proteomic markers while preserving gut morphology, and rescuing behavioral outcomes. Although several nutraceutical and antioxidant compounds have previously been investigated in ASD-related models, studies specifically evaluating cocoa polyphenols within an integrated behavioral and multi-omics framework remain limited. Accordingly, the present study provides a novel framework linking metabolic and neurodevelopmental pathways in a zebrafish model of ASD, while highlighting cocoa polyphenols as a multi-target nutraceutical intervention capable of mitigating VPA-induced metabolic stress at several mechanistic levels.

## Supplementary Information

Below is the link to the electronic supplementary material.

Supplementary material 1 (DOCX 603.7 kb)

## Data Availability

Metabolomic and proteomic datasets are available at https://doi.org/10.5683/SP3/BTGP8V.
