# Supplementary material for: Untargeted metabolomics and proteomics reveals cocoa-mediated mitigation of valproic acid-induced dysregulation in a zebrafish model of autism: pilot study
Source: Metabolomics. 2026 Jun 18;22(4):101. doi: 10.1007/s11306-026-02482-w (PMC13279486; doi:10.1007/s11306-026-02482-w)
Supplement: Supplementary file 1 — Supplementary material 1 (DOCX 603.7 kb) [file 11306_2026_2482_MOESM1_ESM.docx]

**Untargeted Metabolomics and Proteomics Reveals Cocoa-Mediated Mitigation of Valproic Acid-Induced Dysregulation in a Zebrafish Model of Autism: Pilot Study**

Jeffrey Li^1^, Isra’a Haj-Husein^1^, Nathan Ghafari^2^, Leila Khorraminezhad^1^, Michèle Iskandar^1^, Charlotte Zaouter^3^, Matthias Klein^4^, Lekha Sleno^2^, Shunmoogum Patten^3^, Stan Kubow^1^.

Jeffrey Li and Isra’a Haj-Husein should be considered joint first author

^1^ School of Human Nutrition, McGill University, Montreal, Canada

^2^ Département de chimie, Université du Québec à Montréal, Montréal, Canada

^3^ Institut national de la recherche scientifique, Québec City, Canada

^4^ Animal Science, McGill University, Montreal, Canada

**Correspondence**: Stan Kubow ([stan.kubow@mcgill.ca](mailto:stan.kubow@mcgill.ca))

Supplementary Data

1. Methods

***LC-MS/MS for Untargeted Metabolomics***

Chromatographic separation was performed using a pentafluorophenyl (PFP) column (Phenomenex Luna, 2.1 x 150 mm, 3 μm) and the mobile phases consisted of water (0.1% formic acid, FA) and methanol at a flow rate of 250 μL/min. The elution gradient started at 3% B, and was increased to 65% at 15min, up to 85% at 18 min. Metabolite detection was conducted using information-dependent acquisition (IDA) with TOF-MS (*m/z* 80-800) and TOF-MS/MS (*m/z* 40-800) on the top 8 ions per cycle with dynamic exclusion, with a total cycle time of 1.05 s. Data were processed using Markerview 3.0 (Sciex) software for feature generation (*m/z* and retention time). Metabolite identification was performed using the Sciex OS-Q 2.0 software with spectral library matching, applying a library score threshold of >85 and a mass accuracy of protonated or deprotonated molecules <10 ppm.

***LC-MS/MS for Untargeted Proteomics***

Chromatographic separation was performed on an Aeris PEPTIDE XB-C18 column (100 x 2.1 mm, 1.7 μm) with a gradient elution of H_2_O (A) and ACN (B), both containing 0.1% FA, at a flow rate of 300 μL/min. IDA was employed with TOF-MS (*m/z* 140-1250) and TOF-MS/MS (*m/z* 300-1250), targeting the 15 most intense ions per cycle. Data-independent (SWATH) acquisition was also conducted for quantitation with a TOF-MS (*m/z* 140-1250) acquisition, followed by MS/MS (*m/z* 80-1500) with 100 variable precursor windows, and a total cycle time of 2.7 s.

1. Results


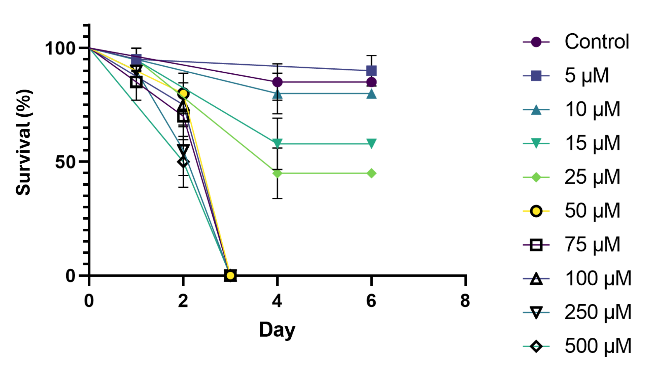


Fig S1 VPA toxicity assessment.

Proportion of larval survival (%) following exposure to increasing concentrations of VPA (5–500 μM). Survival was monitored daily across all treatment groups. Data are presented as mean ± SEM.


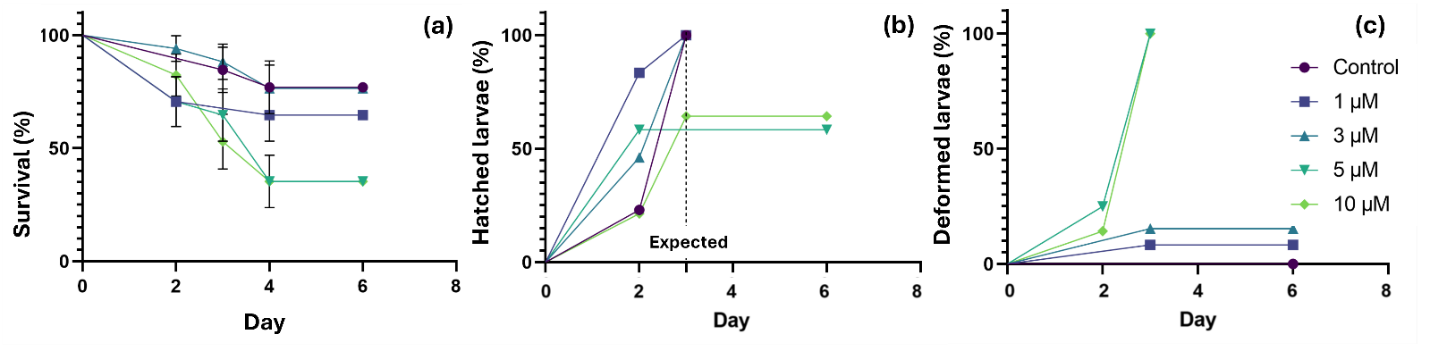


**Fig S2 Effects of VPA exposure on larval survival, hatching, and deformity**

Shown are (a) survival (%) of zebrafish larvae, (b) hatching (%), and (c) deformity (%) following exposure to increasing concentrations of VPA (1–10 μM). Zebrafish larvae are expected to hatch around 3 days post-fertilization (dashed line, “expected”). Data, where applicable, are presented as mean ± SEM.


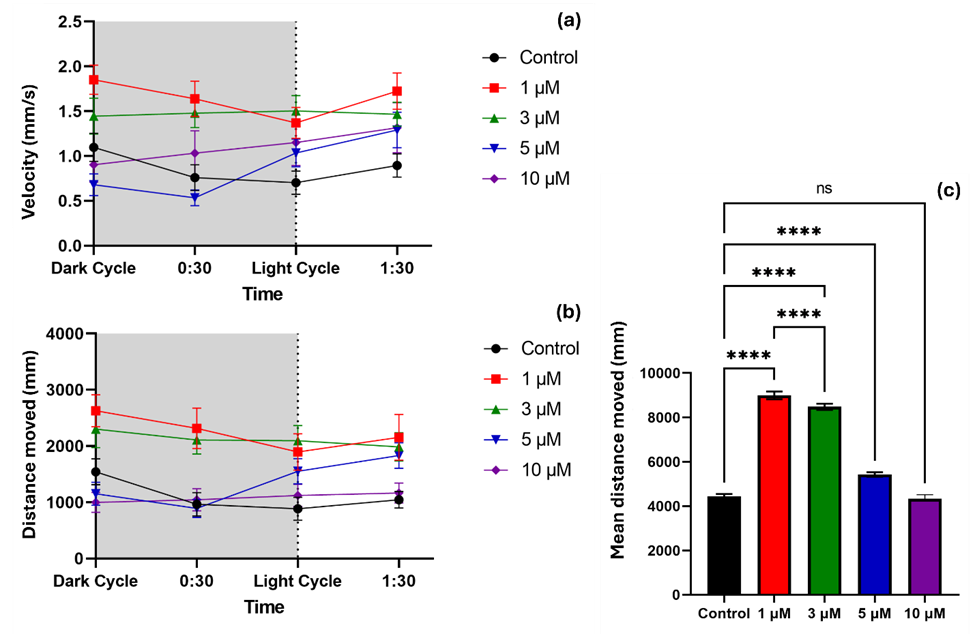


**Fig S3 Behavioral analysis of VPA-treated zebrafish in response to stress-inducing change in environment (dark to light).**

Shown are (a) velocity and (b) distance moved over time across VPA concentration groups (1–10 μM). The shaded region indicates the dark phase, and the transition to the light phase is marked by a dashed line. (c) Mean total distance moved across VPA concentrations. Data are presented as mean ± SEM. Statistical significance is indicated as: ns (not significant) and **** (p < 0.0001).


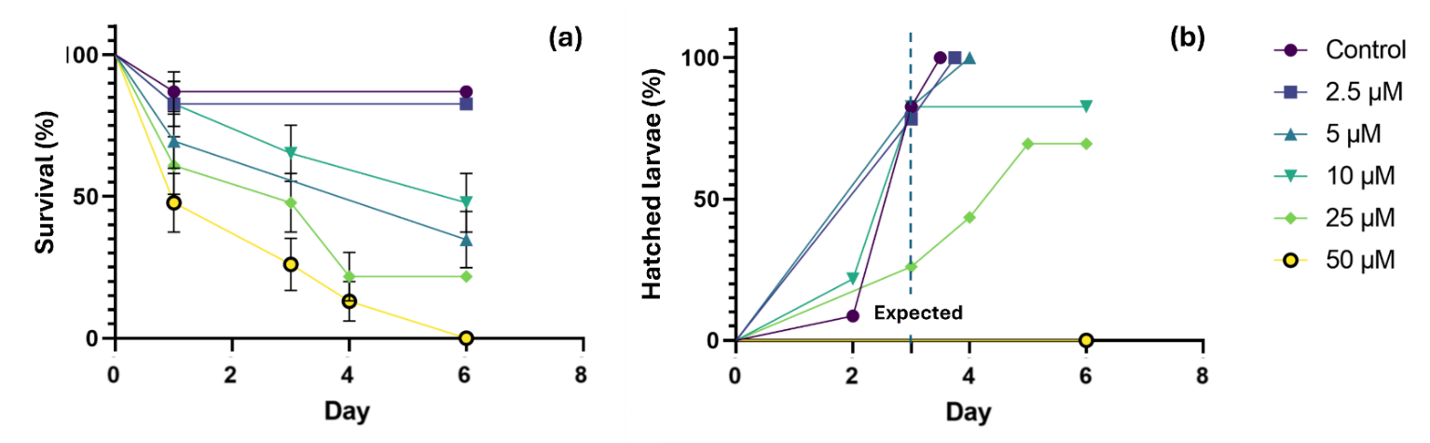


Fig S4 Cocoa powder toxicity assessment.

Shown are (a) survival (%) of zebrafish larvae and (b) hatching (%) following exposure to increasing concentrations of cocoa powder, expressed as (−)-epicatechin equivalents (2.5–50 μM). Zebrafish larvae are expected to hatch around 3 days post-fertilization (dashed line, “expected”). Data, where applicable, are presented as mean ± SEM.


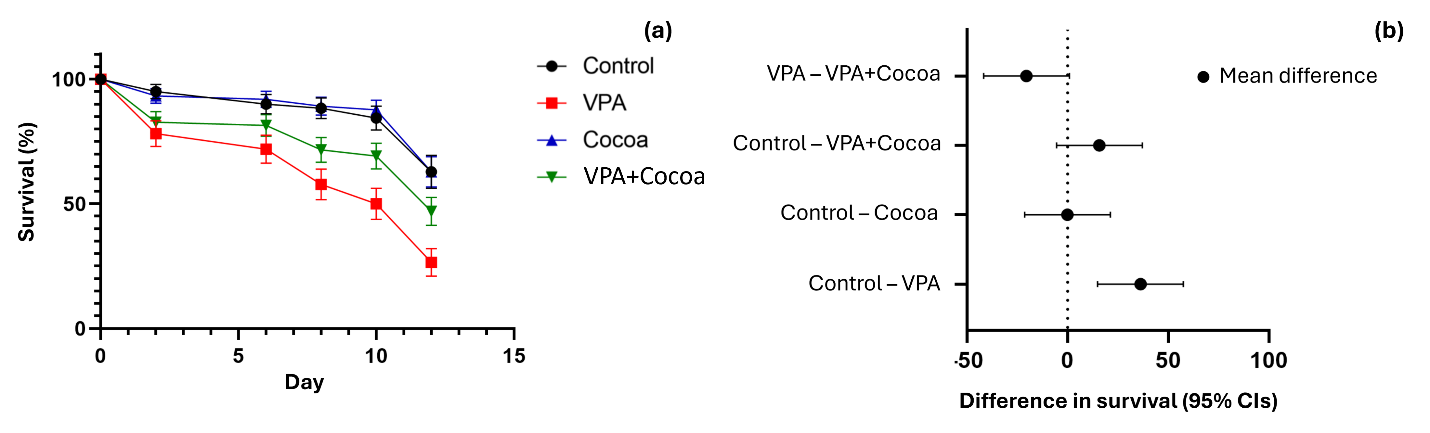


**Fig S5 Survival of zebrafish larvae in response to VPA, cocoa, and co-treatment.**

Shown are (a) larval survival (%) across treatment groups (control, VPA, cocoa, and VPA + cocoa) over 12 days post-fertilization (dpf) and (b) pairwise differences in survival (%) at 12 dpf, presented as mean differences with 95% confidence intervals (CIs) following Bonferroni correction. Data in (a) are presented as mean ± SEM.


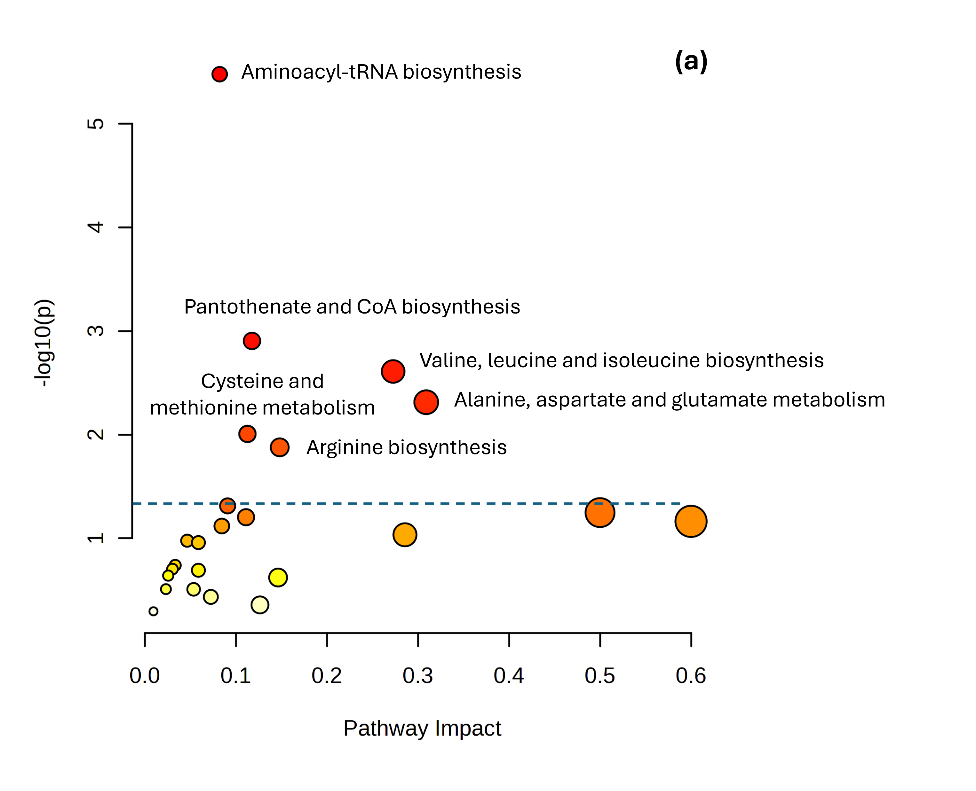


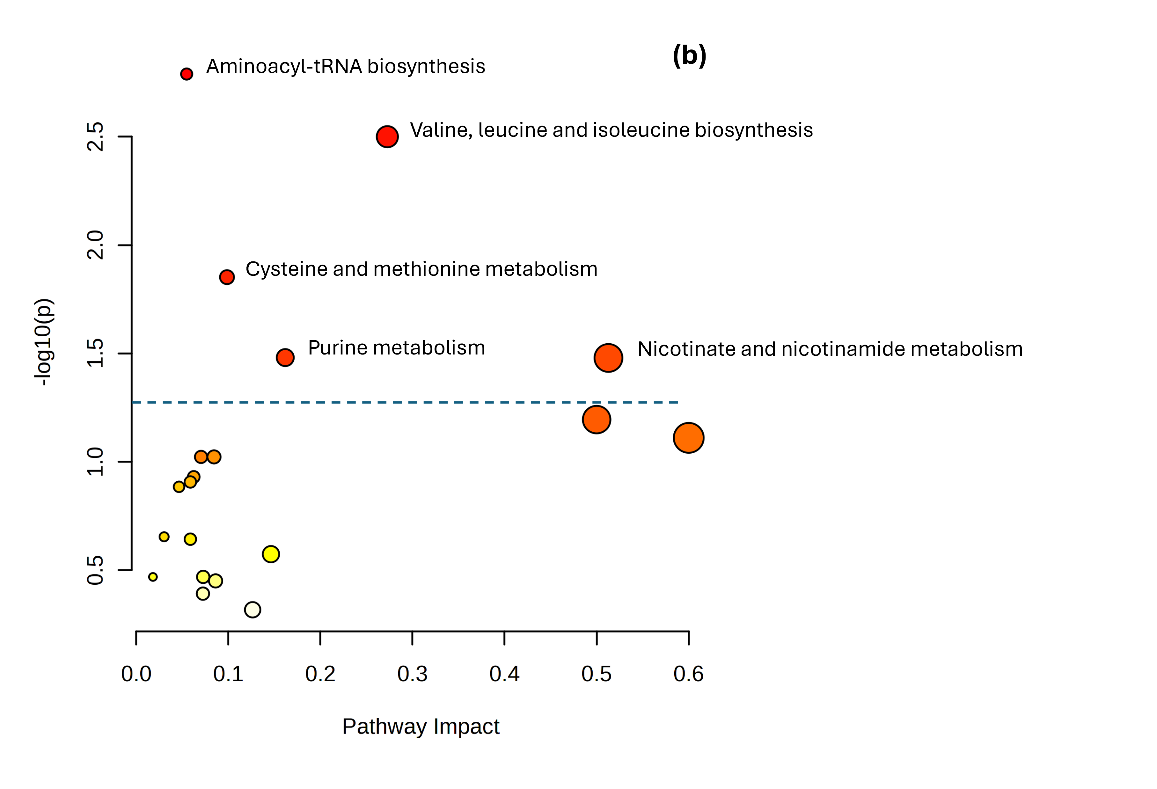


**Fig S6 Joint pathway analysis of metabolomic and proteomic data.**

(a) Pathway enrichment analysis comparing VPA versus control groups. All labeled pathways were significant following false discovery rate (FDR) correction at 20%. (b) Pathway enrichment analysis comparing VPA with cocoa versus VPA groups. Only aminoacyl-tRNA biosynthesis and valine, leucine, and isoleucine biosynthesis remained significant after FDR correction at 20%. The x- and y-axes represent pathway impact and statistical significance, expressed as −log_10_(p), respectively. The dashed line indicates the significance threshold. Circle size reflects pathway impact, and color intensity corresponds to the level of statistical significance.

**Table S1 Differential metabolomic and proteomic features in VPA versus control and VPA with cocoa versus VPA comparisons.**

| **VPA vs. Control** | | |
| --- | --- | --- |
| Metabolite | *p*-value | Log_2_FC |
| O-Phospho-L-Serine | 0.0003 | -1.0 |
| Methionine sulfoxide | 0.001 | 1.1 |
| Urate | 0.003 | 0.4 |
| Hypotaurine | 0.004 | 0.8 |
| Glutamine | 0.009 | -0.3 |
| 5-Methoxytryptophan | 0.010 | 1.9 |
| 6-Hydroxynicotinic acid | 0.019 | 1.1 |
| Tyrosine | 0.021 | 1.1 |
| Citric acid | 0.023 | -0.7 |
| Glycyl-glutamic acid | 0.029 | 1.0 |
| Acetyl-L-Threonine | 0.053 | 0.7 |
| Aminoadipic acid | 0.054 | -0.3 |
| Aspartic acid | 0.054 | -0.2 |
| Isoxanthopterin | 0.057 | 0.4 |
| Valine | 0.064 | 0.7 |
| Cystathionine | 0.069 | 0.7 |
| Flavin Mononucleotide | 0.071 | 0.5 |
| 2-Octenoyl-carnitine | 0.072 | 0.7 |
| Pantothenate | 0.075 | 0.2 |
| Leucine | 0.081 | 0.7 |
| Proline | 0.086 | 0.6 |
| Protein | *p*-value | Log_2_FC |
| Betaine—homocysteine S-methyltransferase 1 | 0.002 | 1.0 |
| Desmoplakin-A | 0.004 | 0.8 |
| Trafficking protein particle complex subunit 11 | 0.031 | 0.6 |
| Proteasomal ubiquitin receptor ADRM1 | 0.047 | 0.6 |
| **VPA + Cocoa vs. VPA** | | |
| Metabolite | *p*-value | Log_2_FC |
| Caffeine | 0.00005 | 4.6 |
| Theobromine | 0.0001 | 5.1 |
| Allantoic acid | 0.001 | -1.6 |
| Methylsuccinic acid | 0.005 | 1.3 |
| Glutathione oxidized | 0.007 | -0.7 |
| Nicotinamide | 0.012 | -0.5 |
| 2-Octenoyl-carnitine | 0.015 | -1.0 |
| Methionine sulfoxide | 0.015 | -0.7 |
| Allantoin | 0.020 | -0.9 |
| Tyrosine | 0.028 | -1.0 |
| Adenosine 5-monophosphate | 0.033 | -0.5 |
| Proline | 0.034 | -0.8 |
| Cystathionine | 0.045 | -0.8 |
| Ophthalmic acid | 0.052 | -0.6 |
| Adenine | 0.054 | -0.4 |
| Leucine | 0.055 | -0.7 |
| NAD | 0.056 | -0.6 |
| 5-Methoxytryptophan | 0.060 | -1.3 |
| Flavin Mononucleotide | 0.061 | -0.6 |
| Citric acid | 0.079 | 0.5 |
| Valine | 0.091 | -0.7 |
| Protein | *p*-value | Log_2_FC |
| Betaine--homocysteine S-methyltransferase 1 | 0.006 | -0.9 |
| Desmoplakin-A | 0.005 | -0.8 |

Metabolites and proteins significantly altered in each comparison are presented with corresponding *p*-values, derived from Tukey’s HSD test, and log_2_ fold changes (log_2_FC). Positive and negative log_2_FC values indicate higher and lower abundance, respectively, in the first group of each comparison. Fold changes were calculated from raw (non-log-transformed) data as the ratio of mean metabolite intensities between groups.
